# The Prognosis and Feasibility of Extensive Clinical Target Volume in Postoperative Radiotherapy for Esophageal Squamous Cell Carcinoma: A Phase II Clinical Trial

**DOI:** 10.3389/fonc.2021.669575

**Published:** 2021-07-02

**Authors:** Xiaofei Zhang, Dashan Ai, Juanqi Wang, Yun Chen, Qi Liu, Jiaying Deng, Huanjun Yang, Yongzhan Nie, Weiwei Chen, Weixin Zhao, Kuaile Zhao

**Affiliations:** ^1^ Department of Radiotherapy, Fudan University Shanghai Cancer Center, Shanghai, China; ^2^ State Key Laboratory of Cancer Biology, Xijing Hospital, Fourth Military Medical University, Xi’an, China; ^3^ Third People’s Hospital, The Sixth Affiliated Hospital of Nantong University, Yancheng Hospital Affiliated Southeast University Medical College, Yancheng, China

**Keywords:** esophageal squamous cell carcinoma, IMRT, radiotherapy, postoperative treatment, CTV

## Abstract

**Background:**

This trial aims to explore the feasibility and safety of postoperative radiotherapy covering all regional lymph node areas for locally advanced thoracic esophageal squamous cell carcinoma patients treated with intensity-modulated radiation therapy (IMRT).

**Methods:**

This was a single-center single-arm, phase II clinical trial initiated in 2014. Patients who were treated with radical transthoracic resection and had negative margins within 3 months and histologically confirmed esophageal squamous cell carcinoma (pT3-4 or N+, M0 determined by the 7th edition of the AJCC guidelines) were recruited in this trial. Postoperative radiotherapy was performed with a total dose of 40 Gy in 20 fractions using IMRT. Clinical target volumes (CTVs) included the tumor bed, anastomosis, bilateral supraclavicular region, mediastinal lymph nodes, left gastric lymph nodes and celiac trunk lymph nodes. The primary endpoint was the 2-year local control rate, and the secondary endpoints were overall survival (OS) and adverse events (AEs).

**Results:**

A total of 70 eligible patients were recruited from 2014 to 2016. The 2-year local control rate, as the primary endpoint, was 67.3%. In addition, the median OS was 57.0 months, with 1-year and 3-year OS rates of 92.8% and 60.9%, respectively. Among the patients, 28/40 (40%) developed locoregional recurrence, with 25.7% involving hematogenous recurrences. All reported AEs occurred during the course of IMRT or within 6 months thereafter. None of them suffered grade 4 hematological or nonhematological AEs. Nearly all patients completed the entire course of postoperative radiotherapy, with a completion rate of 97.1%.

**Conclusion:**

For an extensive target volume, 40 Gy is feasible and shows acceptable toxicity in patients with locally advanced thoracic esophageal squamous cell carcinoma, although the local recurrence rate is relatively high. Our findings provide a basis for further exploration of high-dose radiation with extensive CTV combined with chemotherapy.

**Clinical Trial Registration:**

[http://www.clinicaltrials.gov/ct2/results?cond=&term=NCT02384811&cntry=&state=&city=&dist=], identifier [NCT02384811].

## Introduction

Esophageal carcinoma is an aggressive tumor and the leading cause of cancer-related death worldwide. It is frequently detected in China, where it ranks third in cancer-related mortality (1). Surgery is preferred for esophageal cancer patients with operative indications. However, the 5-year survival of esophageal carcinoma patients treated with surgery only is only 20 - 30% (2), which is mainly attributed to local recurrence and lymph node metastasis (3). Theoretically, postoperative radiotherapy can kill residual and subclinical lesions in esophageal cancer patients, which significantly reduces the recurrence rate and prolongs survival.

In recent years, a growing number of clinical trials and large-sample retrospective studies have demonstrated that postoperative radiotherapy can improve the local control rate and prolong survival in advanced esophageal cancer patients (T3/T4 N+, M0) (4–7). Xiao et al. reported that among stage III patients (T4N0-1M0 or T3N1M0 determined by the 7^th^ edition of the AJCC guidelines), those in the postoperative radiotherapy group had a higher 5-year survival rate than those in the surgery-only group (4, 5). Therefore, the Chinese Society of Clinical Oncology (CSCO) guidelines recommend postoperative radiotherapy for patients with locally advanced esophageal cancer (T3/4 or N+, M0) (8).

The incidence of lymph node metastasis among esophageal cancer patients is relatively high and exhibits a scattered pattern. Theoretically, all high-risk lymphatic drainage areas should be covered during radiotherapy, leading to large irradiation volumes and high toxicity. As a result, a variety of target area designs have been proposed. In Xiao et al.’s report (4, 5), the entire mediastinum involving bilateral supraclavicular areas and the site of anastomosis were set as target volumes. Postoperative radiotherapy therefore significantly reduces recurrence in the supraclavicular and mediastinal lymph nodes, but recurrence in abdominal lymph nodes remains high, which is largely attributed to the uninvolved abdominal lymph nodes in the irradiation field. Based on the above conclusions, we believe that within normal tissue dosing constraints, all high-risk lymph node drainage areas should be preventively irradiated and that the irradiation volume in different parts of esophageal cancer lesions should remain the same. In this clinical trial, CTVs included the tumor bed if preoperative CT images were available, as well as the anastomosis, bilateral supraclavicular lymph nodes, all mediastinal lymph nodes, left gastric lymph nodes and celiac lymph nodes.

Nonetheless, different opinions have arisen in the literature, including the notion that postoperative radiotherapy for esophageal cancer does not improve survival (9–11). These studies were conducted as early as the 1990s or even remotely and may be biased by different recruitment standards, long recruitment durations, low-quality two-dimensional radiotherapy equipment, and inconsistent dose requirements. Two-dimensional technology was once widely adopted in research into extensive target volume irradiation, but its protective capacity on normal tissues was found to be significantly lower than that of intensity-modulated radiotherapy (IMRT) (12). Thus, in our clinical trial, IMRT technology was adopted for extensive target volume irradiation, which was based on computer planning studies, as it yielded better planning target volume (PTV) coverage and better spared organs at risk.

This single-center, single-arm, phase II clinical trial study aims to explore the feasibility and safety of postoperative radiotherapy on extensive CTVs in patients with locally advanced esophageal cancer (T3-4 or N+, M0) using the IMRT radiation technique. We assumed that all high-risk lymph node drainage areas within the normal tissue dose constraints would be preventively irradiated and that the irradiation volume of different parts in esophageal cancer lesions would remain the same. CTVs included the tumor bed if preoperative CT images were available, as well as the anastomosis site, bilateral supraclavicular lymph nodes, all mediastinal lymph nodes, left gastric lymph nodes and celiac lymph node area. A postoperative prophylactic irradiation dose of 40 Gy was adopted as a safe dose.

## Materials and Methods

### Study Design

This trial was a single-center single-arm, phase II clinical trial (NCT02384811) conducted from 2014 to 2016 at Fudan University Shanghai Cancer Center. Patients who were treated with radical transthoracic resection and had negative margins within 3 months and histologically confirmed esophageal squamous cell carcinoma (pT3-4 or N+, M0 determined by the 7^th^ edition of the AJCC guidelines) were eligible. Tumor size and extent were coded primarily from the operative report and pathology reports and therefore likely representative of pathologic staging. The extent of nodal disease was determined based on pathologic findings only. This information was used to convert the extent of disease to tumor, node, and metastasis staging according to the 7^th^ edition of the AJCC guidelines. Preoperative examination included CT scan of the thorax, ultrasound from the supraclavicular region to the abdomen, esophagography and gastroscopy. None of the patients received preoperative or postoperative radiotherapy and/or chemotherapy prior to recruitment. No locoregional recurrent disease or distant metastases were found before postoperative radiotherapy. The inclusion criteria were as follows: age < 75 years; Karnofsky Performance Status score ≥80; neutrophil count ≥ 1.5 × 10^9^/L; leukocyte count ≥ 3 × 10^9^/L; platelet count ≥100 × 10^9^/L; serum creatinine (SCr) level < 1.5 upper limit of normal (ULN); and alanine aminotransferase (ALT) or aspartate transaminase (AST) < 2.5 ULN. All participants provided written informed consent.

### Intervention

**Figure 1:**
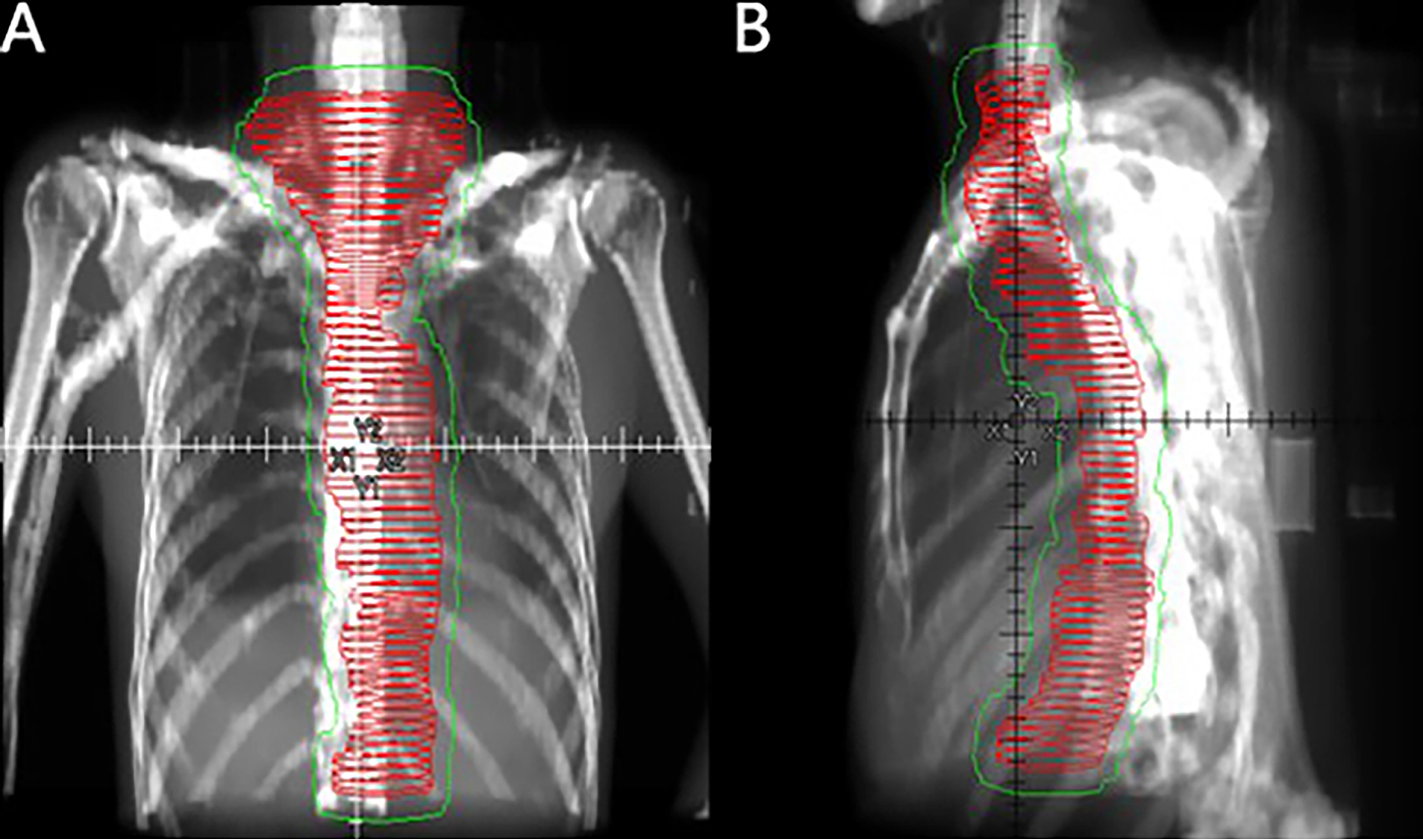
Coronal **(A)** and sagittal **(B)** views of the CTV (red horizontal lines) and PTV (thin green line) of large-field postoperative radiotherapy using the three-dimensional radiation technique.

Radiotherapy was initiated within 3 months after surgery using 6-MeV photons delivered by a linear accelerator for a total dose of 40 Gy in 20 fractions using IMRT. Radiotherapy was delivered after the patient underwent surgery. Patients were treated 5 days per week at 2.0 Gy/d. Target volumes were defined in accordance with the 1999 ICRU Report #62 (13), and the defined CTVs included the tumor bed, anastomosis site, bilateral supraclavicular region, all mediastinal lymph node sites, and left gastric and celiac trunk lymph nodes. The PTV provided proximal, distal, and radial margins of 1 cm around the CTV. The field next to the spinal cord could be slightly adjusted to reduce exposure (**Figure 1**). When formulating the treatment plan, normal organ dose limitations were taken into consideration, as described in **Supplemental Table 1**. The 95% isodose had to encompass the entire PTV. The maximum dose delivered to the PTV did not exceed the prescription dose by 10%. Tissue density inhomogeneity correction was used. The patients were followed-up at least once a week during the courses of postoperative radiotherapy to monitor AEs. Radiotherapy was delayed when one of the following conditions were met: WBC<2.0×10^9^/L; ANC<1.0×10^9^/L; PLT<50×10^9^/L; or grade 3 or higher nonhematological toxicity. Treatment was suspended until the toxicity had resolved to grade 2 or lower. In addition, radiotherapy was delayed for patients with mediastinal or thoracic infection with a fever over 38.5°C until they completely recovered. A maximum 2-week suspension to lower toxicity or to allow for complete recovery of infection was permitted; otherwise, radiotherapy was terminated. After finishing the entire treatment, patients were followed-up for local recurrence and distant metastasis every 3 months within the first year, every 6 months in the next 2 years, and once a year thereafter. Follow-up visits consisted of disclosure of new complaints and physical examinations such as palpation of supraclavicular lymph nodes. Diagnostic tests including CT scan of the thorax and neck, ultrasound from the supraclavicular region to the abdomen, and esophagography were requested as clinically indicated. Gastroscopy was performed once a year.

### Outcomes

The primary endpoint was the 2-year local control rate in all recruited patients, in which local control was defined as recurrence in the esophageal stump or lymph nodes in the radiation field. The secondary endpoints involved overall survival (OS) and toxicity. OS was defined as the time from the date of surgery until death. Toxicity was evaluated according to the National Cancer Institute Common Terminology Criteria for Adverse Events (AE) (NCI-CTCAE 4.0). Disease-free survival (DFS) was defined as the time from the date of surgery to the date of recurrence, metastasis or death, whichever occurred first. Metastasis-free survival (MFS) was defined as the time from the date of surgery until the day of recurrence at any site that exceeded the primary tumor region and regional lymph nodes within the irradiation field or death due to any reason. Locoregional recurrence-free survival (LRFS) was defined as the time from the date of surgery until the date of recurrence in the esophageal stump or lymph nodes within the irradiation field or death due to any reason.

### Statistical Analysis

In the CROSS clinical trial, the 2-year local recurrence rates in the neoadjuvant radiotherapy and chemotherapy + surgery group and surgery-only group were 14% and 34%, respectively (14). Therefore, we tested an inferior 2-year local recurrence rate in the adjuvant radiotherapy + surgery group versus the surgery-only group. The necessary sample size to guarantee an improvement of 20% in the 2-year local recurrence rate, with a global alpha risk of 5%, power of 80%, an accrual period of 18 months and 10% patient loss, was calculated. Survival was estimated using the Kaplan-Meier method. SPSS 22.0 was used for data analyses.

## Results

### Clinical Characteristics

A total of 70 eligible patients were recruited for this phase II study from 2014 to 2016, and their clinical characteristics are listed in **Table 1**. The majority were male (90.0%), and the average age of the recruited patients was 60 years (43-73 years). The median tumor length was 3 cm (2-12.7 cm). Two-field lymphadenectomy was performed in 51/70 (72.9%) patients. The average number of lymph nodes removed was 25.9. In terms of tumor stage, stage IIa (40%), IIIA (27.1%) and IIIB (27.1%) were the most frequently examined. Most tumors were located in the middle (37.1%) and lower (51.4%) regions.

**Table 1:** Patient Characteristics (N = 70).

Characteristics	N (%)
Age (y)	
Median(range)	60 (43-73)*
Sex	
Male	63 (90.0)
Female	7 (10.0)
Smoking history	
Never	20 (28.6)
Former or current	50 (71.4)
Drinking history	
Never	22 (31.4)
Former or current	48 (68.6)
Stage (AJCC, 6th edition)	
IIa**	28 (40)
IIb	0 (0)
IIIa	19 (27.1)
IIIb	19 (27.1)
IIIc	4 (5.7)
T phase	
T2	3 (4.3)
T3	66 (94.3)
T4a	1 (1.4)
N phase	
N0	28 (40.0)
N1	20 (28.6)
N2	19 (27.1)
N3	3 (4.3)
Tumor location	
Upper (<25cm)	7 (10.0)
Middle (25-30cm)	26 (37.1)
Lower (>30cm)	36 (51.4)
Multi-primary	1 (1.4)
Lymphadenectomy	
Two-field	51 (72.9)
Three-field	10 (14.3)
unknown	9 (12.9)
Histologic differentiation	
Poor	11 (15.7)
Poor-Medium	13 (18.6)
Medium	34 (48.6)
Medium-Well	8 (11.4)
Well	1 (1.4)
Unknown	3 (4.3)

*The unit is years old.

**Stage IIA patients is T3N0.

### Treatment

**Figure 2:**
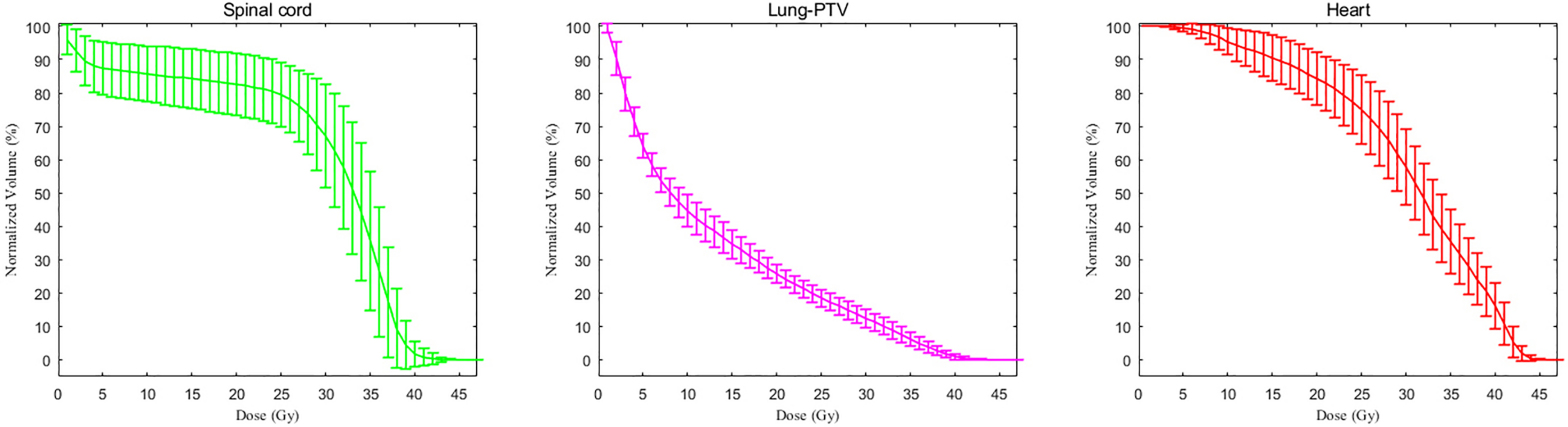
The dose-volume histogram parameters of normal tissue and organs. The dose-volume histogram curve comprises the average values, and the upper and lower ranges represent the standard deviations.

Radiotherapy parameters are listed in **Table 2**. The average PTV dose for all patients was 39.5 ± 3.7 GY, and the median PTV dose was 40 GY. The median tumor volume was 1273.2 cm^3^. The median values of the average dose, V5, and V20 of the lungs were 13.0 Gy, 64.6% and 26.2%, respectively. The median value of the maximum dose delivered to the spinal cord was 40.9 Gy. For the heart, the median value of the average dose delivered and the V30 were 30.3 Gy and 60.0%, respectively; for the liver, they were 11.7 Gy and 12.7%, respectively. The median value of the average dose and V15 of the kidney were 9.5 Gy and 21%, respectively. Although the target volume was relatively large, the dose-volume histogram (DVH) parameters of normal tissues were relatively ideal, although the average dose to the heart was slightly higher than the dose-volume constraints recommended by the NCCN (**Figure 2**). Referring to the dose-volume constraints recommended in our trial (**Supplemental Table 1**), the exceedance rates of the maximum dose to the spinal cord, V20 of the lung, V5 of the lung, average dose delivered to the lung, V30 of the heart, and average dose delivered to the heart were 6.5%, 71.7%, 52.2%, 10.9%, and 87.0%, respectively. Nearly all patients completed the entire course of postoperative radiotherapy, with a completion rate of 97.1%. Two of the 70 patients did not complete the trial, with one patient having grade 3 radiation-induced pneumonitis and the other having a poor performance status.

**Table 2:** Radiotherapy Parameters.

Radiotherapy parameters	
Dose,Gy	40.0
Completed full dose of planning radiotherapy, %	68.0 (97.1)
PTV, cm3	1273.2 [1023.2,1560.0]*
Mean lung dose, Gy**	13.0 [11.3,14.4]
Lung V5, %	64.6 [56.5,71.9]
Lung V20, %	26.2 [19.9,30.8]
Spinal cord maximum dose, Gy	40.9 [38.2,45.6]
Mean heart dose, Gy	30.3 [25.3,34.8]
Heart V30, %	59.9 [42.2,84.1]
Mean liver dose, Gy	11.7 [8.7,16.1]
Liver V30, %	12.7 [5.6,29.1]
Mean kidney dose, Gy	9.5 [5.1,28.1]
Kidney V15, %	21.0 [14.0,85.0]

*Median (95% confidence interval).

**Lung-PTV was used for lung dose assessment.

### Survival and Treatment Failure Pattern

**Figure 3:**
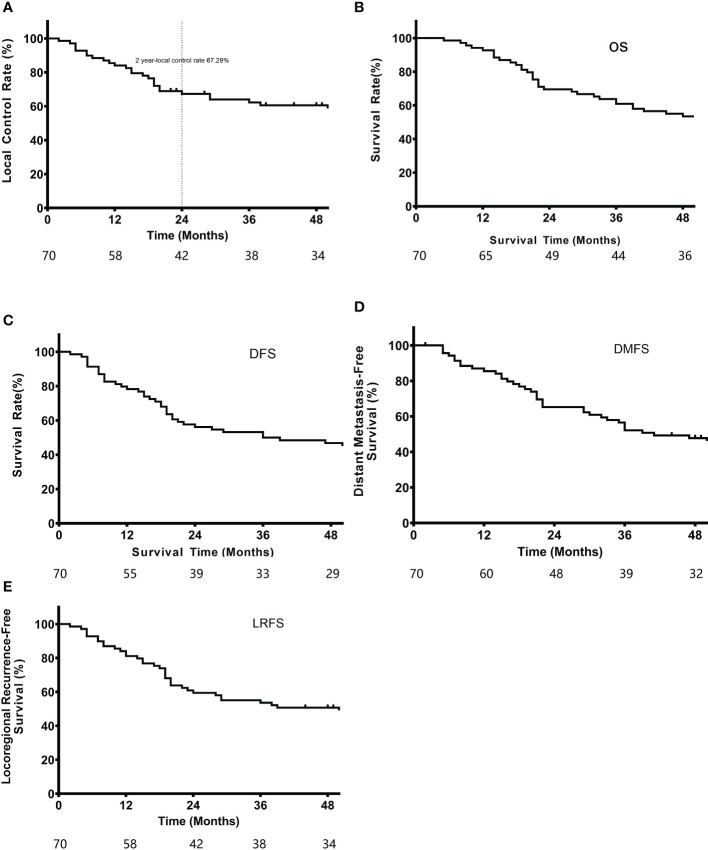
**(A)** Local control rate, **(B)** OS, **(C)** DFS, **(D)** MFS, and **(E)** LRFS of the enrolled patients.

A total of 70 patients recruited from 2014 to 2016 were followed-up until February 6, 2020. The median follow-up duration was 48 months (2-72 months). Seventeen patients died during the follow-up period. The 2-year local control rate, which was the primary endpoint, was 67.3%. The median LRFS was 39.0 months, and the 1-year and 3-year LRFS rates were 81.2% and 53.6%, respectively. In addition, the median OS was 57.0 months, and the 1-year and 3-year OS rates were 92.8% and 60.9%, respectively. The median DFS was 36.0 months, with 1-year and 3-year DFS rates of 78.3% and 50.0%, respectively. The median DMFS was 46.2 months, including 1-year and 3-year DMFS rates of 85.5% and 52.2%, respectively (**Figure 3**).

We further analyzed the patterns of treatment failure, as shown in **Table 3**. Initial tumor recurrence was the major cause of treatment failure, with 28 (40%) patients experiencing locoregional recurrence [mostly in the mediastinal lymph nodes, involving 13 in the middle upper mediastinum (2-7 zones) and 3 in the lower mediastinum (8 and 9 zones)]. In addition, 10 (14.3%) patients developed locoregional recurrence in the supraclavicular lymph nodes, 5 (7.1%) in the anastomosis site, and 3 (4.3%) in the celiac lymph nodes. In addition, 18 (25.7%) patients experienced hematogenous recurrence. The lung was the most frequently metastasized organ, followed by the bone and the liver.

**Table 3:** Patterns of First Treatment FailureΔ.

First Failure	N (%)*
No Failure	32 (45.7%)
Loco-regional Failure (In field)**	28 (40.0%)
Anastomosis	5 (7.14)
Supraclavicular Lymph Node	10 (14.28)
Mediastinal Lymph Node	16 (22.86)
Celiac Lymph Node	3 (4.29)
Distant Metastasis	18 (25.7%)
Lymph node outside radiation field	1 (1.43)
Lung	10 (14.29)
Liver	5 (7.14)
Bone	6 (8.57)
Pleura	1 (1.42)
Secondary primary tumor***	2 (2.86)
Base of tongue	1 (1.43)
Colon	1 (1.43)

ΔConcurrent recurrence is defined as different recurrences within 2 months.

*There will be overlapping of patients in various recurrence situations, but the denominator is 70 when we calculating the ratio.

**Because some preoperative CT images can't be obtained, the data of anastomotic stoma can't be obtained.

***Second primary tumor excludes the second primary of esophagus.

### Toxicity

All AEs were reported and are listed in **Table 4**, among which grade 3 or higher AEs occurred in more than 10% of patients. Severe hematological and nonhematological toxicities were rarely reported in this trial. All AEs occurred during or within 6 months after radiotherapy. No long-term AEs occurred. Four (5.7%) patients developed acute grade 3 leukopenia, and 1 (1.4%) had neutropenia. Among the reported acute grade 3 nonhematological toxicities, nausea and vomiting (7.1%) were the most frequent AEs, followed by fatigue (2.8%) and radiation-induced pneumonitis (1.4%). None of the patients developed grade 4 hematological or nonhematological AEs.

**Table 4:** Acute Treatment Toxicity.

Toxicity	N (%)*				
	Grade 0	Grade 1	Grade 2	Grade 3	Grade 4
Hematological toxicity					
Leukopenia	11 (15.7)	31 (44.3)	24 (34.3)	4 (5.7)	0 (0)
Neutropenia	52 (74.3)	12 (17.1)	5 (7.1)	1 (1.4)	0 (0)
Anemia	56 (80.0)	14 (20.0)	0 (0)	0 (0)	0 (0)
Thrombocytopenia	35 (50.0)	30 (42.9)	5 (7.1)	0 (0)	0 (0)
Nonhematological toxicity					
Fatigue	48 (68.6)	14 (20.0)	6 (8.6)	2 (2.8)	0 (0)
Nausea/vomiting	24 (34.3)	32 (45.7)	9 (12.9)	5 (7.1)	0 (0)
Esophagitis	26 (37.2)	39 (55.7)	5 (7.1)	0 (0)	0 (0)
Pneumonitis	29 (41.4)	27 (38.6)	13 (18.6)	1 (1.4)	0 (0)
Dermatitis	64 (91.4)	5 (7.1)	1 (1.4)	0 (0)	0 (0)
hydropericardium	54 (77.1)	15 (21.4)	1 (1.4)	0 (0)	0 (0)

Acute AE were defined as occurred during or within 6 months after radiotherapy.

*The denominator is 70 when we calculating the ratio.

## Discussion

This was the first phase II clinical trial to explore the effect and safety of postoperative radiotherapy for extensive target volumes that cover all regional lymph nodes, as defined in the 7^th^ edition of the AJCC/UICC guidelines in patients with locally advanced thoracic esophageal squamous cell carcinoma using a three-dimensional IMRT technique with a prescribed dose of 40 Gy. Compared with previous studies, our prospective study is superior in terms of the highly matched characteristics of the included population, the use of IMRT radiotherapy equipment, and the consistency of the prescribed dose and target volume.

To date, only a few studies have explored preventative dose prescriptions of postoperative radiation for esophageal cancer. Fletcher et al. reported that a 50-Gy dose can control 90% of subclinical lesions in patients with gastrointestinal tumors (15). The results of RTOG 94-05 showed that compared with a 50.4-Gy dose, a 64.8-Gy dose did not improve survival in esophageal cancer patients undergoing radical radiotherapy and chemotherapy (16). Hence, the conventional dose of 50.4 Gy has been recommended for radical radiotherapy. In RTOG 8501 (17), the dose to the prophylactic irradiation area is 30 Gy during concurrent chemotherapy. Therefore, considering safety, a dose of 40 Gy was adopted in this trial for postoperative prophylactic irradiation. The only similar study to date (5) indicated that postoperative radiotherapy with a prescribed dose of 50-60 Gy involving the entire mediastinum, anastomosis site, and left epiploic and paracardiac lymphatics using two-dimensional radiotherapy technology could significantly improve radiotherapy efficacy on stage III esophageal cancer. In comparison, our CTV included the abovementioned target volume as well as the celiac lymph nodes. Although the prescribed dose of 40 Gy used in our trial was relatively lower than the conventional dose of 50 Gy according to the calculations in **Supplemental Table 2**, the dose to the heart was relatively high. Therefore, a dose of 50-60 Gy is not feasible for such an extensive target volume.

In our study, the 2-year local control rate was low. Moreover, the local recurrence rate was higher than that in the CROSS study (17), which was used to calculate the sample size. We considered that patients in the preoperative radiotherapy group in the CROSS study were treated with concurrent chemotherapy, and adenocarcinoma accounted for 75% of patients, which may have resulted in these differences.

As shown in **Supplemental Table 3**, the percentage of hematogenous metastasis was similar to that in Xiao’s study, but intrathoracic and supraclavicular lymph node recurrence occurred more frequently in our study (4). The following reasons may contribute to the differences. First, the tumor stage of the recruited patients was more advanced in our trial. In our study, two-field lymphadenectomy (72.9%) was the predominant surgical procedure, and patients with supraclavicular lymph node metastasis may be underestimated. Second, compared with the prescribed dose of 50-60 Gy in previous studies (4–7), the dose of 40 Gy used in our trial was relatively low considering the lack of chemotherapy. Thus, it is necessary to increase the dose. Only 3 patients (4.29%) in our trial had celiac lymph node metastasis, suggesting that the extensive target volume we used could largely reduce recurrence in abdominal lymph nodes.

In Zhang et al.’s study (6), the median time to recurrence was 9.56 months in the surgery-alone group and 20.07 months in the surgery+radiotherapy group, with hematogenous recurrence developing after 8.838 and 14.456 months, respectively. Here, the median LRFS and DMFS were 39 and 46.19 months, respectively, which were superior to those reported by Zhang et al. (6). Although our extensive target volume irradiation dose of 40 Gy did not significantly reduce the local recurrence rate, the time to local or hematogenous recurrence was prolonged. As shown in **Figure 3A**, most cases of local recurrence occurred by 2 years, and the curve was relatively stable, suggesting that local recurrence most likely developed 2 years after resection/radiation.

The secondary endpoint results for OS were ideal. Indeed, the observed 3-year OS of 60.87% in this study was significantly better than the 28.9% reported in Schreiber’s study using the SEER database, which included patients with stage III esophageal carcinoma (T3N1M0 or T4N0-1M0) of both squamous and adenocarcinoma origins (18). Moreover, the 1-year, 2-year, 3-year, and 4-year OS and DFS rates in this study were comparatively better than those in previous studies involving stage IIb/III patients with different radiotherapy target areas and a radiotherapy dose of 60 Gy (4, 5). Overall, our postoperative radiotherapy mode was beneficial for improving OS.

We further explored the feasibility of implementing extensive target volume irradiation. Although the mean dose to the heart was relatively high, the doses to the normal tissues in the lungs, spinal cord, liver, and kidney were acceptable. Moreover, the complete radiotherapy compliance rate in our trial was substantially high (97.1%), which may be attributed to improvements in radiotherapy techniques, staging methods, and supportive care.

Our study showed the acceptable safety of extensive target volume irradiation. None of the patients experienced grade 4 hematological or nonhematological toxicity, and the incidences of acute grade 3 hematological and nonhematological toxicity were acceptable. The most frequent AEs were leukopenia and radiation pneumonia, consistent with previous studies (4–7). Nausea and vomiting were reported, which were mainly due to the extensive target volume.

Several limitations should be considered when interpreting our findings. First, this was a single-arm study lacking a control group. Our results were compared with previously published data, which may cause potential biases because of the different time points, places and patient conditions across studies. Second, some recruited patients had a history of surgery at other hospitals, and their medical history was not sufficiently detailed. For example, as preoperative CT could not be obtained for all patients, it was impossible to delineate the tumor bed for every patient. Third, we used the cross study which is a representative prospective study in the treatment of esophageal cancer with chemotherapy plus surgery group and surgery group. At that time, we did not select a suitable representative study for prospective treatment of esophageal cancer.

We did not include postoperative chemotherapy in this trial, which was based on the results of the JCOG9204 study (19), in which the 5-year OS of patients with esophageal squamous cell carcinoma did not benefit from surgery plus chemotherapy or surgery alone. Few studies have compared postoperative chemoradiotherapy with radiotherapy alone. However, the distant metastasis rate and the local recurrence rate were relatively high in our trial, as mentioned above. Postoperative radiotherapy plus chemotherapy is also considered an effective way to improve the efficacy. In conclusion, our radiation field was feasible and reasonable, but the dose needs to be increased. Another clinical trial using extensive target volume irradiation with a dose of 45 Gy plus concurrent chemotherapy with a regimen of paclitaxel plus carboplatin has been launched by our research team (NCT02916511), in which patient recruitment has just been completed.

## Conclusions

This is the first phase II study on postoperative radiotherapy for locally advanced esophageal cancer in the three-dimensional era. The results showed that 99% of lymph node recurrences occurred in the radiation field, indicating that the radiation volume was sufficient. However, the rate of local recurrence in the radiotherapy group was not significantly improved compared with that in the operation group, indicating that the radiation dose should be increased. Notably, the dose distribution for extensive target volume irradiation was feasible and showed acceptable toxicity. This study provides a basis for the further exploration of high-dose extensive target volume irradiation and combined chemotherapy strategies.

## Data Availability Statement

The raw data supporting the conclusions of this article will be made available by the authors, without undue reservation.

## Author Contributions

XZ and DA analyzed the data and were major contributor in writing the manuscript. KZ, WZ, and WC were major contributor to the trial design and the enrollment of patients. JW, YC, QL, DJ, HY, and YN is responsible for the enrollment, efficacy and safety records of the patients. All authors contributed to the article and approved the submitted version.

## Funding

This study was financially supported by the National Natural Science Foundation of China Research, China (grant number: 21172043, 21441010, 81872454), and National Key R&D Program of China (Grant No.2016YFC1303200).

## Conflict of Interest

The authors declare that the research was conducted in the absence of any commercial or financial relationships that could be construed as a potential conflict of interest.

## References

[1] Chen W Zheng R Baade PD Zhang S Zeng H Bray F. Cancer Statistics in China, 2015. CA Cancer J Clin (2016) 66:115–32. 10.3322/caac.21338 26808342

[2] Nakagawa S Kanda T Kosugi S Manabu O Tsutomu S Katsuyoshi H. Recurrence Pattern of Squamous Cell Carcinoma of the Thoracic Esophagus After Extended Radical Esophagectomy With Three-Field Lymphadenectomy. J Am Coll Surg (2004) 198:205–11. 10.1016/j.jamcollsurg.2003.10.005 14759776

[3] Bhansali MS Fujita H Kakegawa T Yamana H Ono T Hikita S. Pattern of Recurrence After Extended Radical Esophagectomy With Three-Field Lymph Node Dissection for Squamous Cell Carcinoma in the Thoracic Esophagus. World J Surg (1997) 21:275–81. 10.1007/s002689900228 9015170

[4] Xiao ZF Yang ZY Liang J Miao YJ Wang M Yi WB. Value of Radiotherapy After Radical Surgery for Esophageal Carcinoma: A Report of 495 Patients. Ann Thorac Surg (2003) 75:331–6. 10.1016/S0003-4975(02)04401-6 12607634

[5] Xiao ZF Yang ZY Miao YJ Wang LH Yin WB Gu XZ. Influence of Number of Metastatic Lymph Nodes on Survival of Curative Resected Thoracic Esophageal Cancer Patients and Value of Radiotherapy: Report of 549 Cases. Int J Radiat Oncol Biol Phys (2005) 62:82–90. 10.1016/j.ijrobp.2004.08.046 15850906

[6] Zhang W Liu X Xiao Z Zhang H Chen D Feng Q. Postoperative Intensity-Modulated Radiotherapy Improved Survival in Lymph Node-Positive or Stage III Thoracic Esophageal Squamous Cell Carcinoma. Oncol Res Treat (2015) 38(3):97–102. 10.1159/000375391 25792080

[7] Chen J Pan J Zheng X Zhu K Li J Chen M. Number and Location of Positive Nodes, Postoperative Radiotherapy and Survival After Esophagectomy With Three-Field Lymph Node Dissection for Thoracic Esophageal Squamous Cell Carcinoma. Int J Radiat Oncol Biol Phys (2012) 82(1):475–82. 10.1016/j.ijrobp.2010.08.037 20934269

[8] Wang FH Shen L Li J Zhou Z-W Liang H Zhang X-T. The Chinese Society of Clinical Oncology (CSCO): Clinical Guidelines for the Diagnosis and Treatment of Gastric Cancer. Cancer Commun (2019) 39(1):10. 10.1186/s40880-019-0349-9 PMC6423835 30885279

[9] Teniere P Hay JM Fingerhut A Fagniez PL. Postoperative Radiation Therapy Does Not Increase Survival After Curative Resection for Squamous Cell Carcinoma of the Middle and Lower Esophagus as Shown by a Multicenter Controlled Trial. French University Association for Surgical Research. Surg Gynecol Obstet (1991) 173:123–30. 10.1016/0005-2787(74)90198-1 1925862

[10] Yamamoto M Yamashita T Matsubara T Kitahara T Sekiguchi K Furukawa M. Reevaluation of Postoperative Radiotherapy for Thoracic Esophageal Carcinoma. Int J Radiat Oncol Biol Phys (1997) 37:75–8. 10.1016/S0360-3016(96)00473-7 9054879

[11] Bedard EL Inculet RI Malthaner RA Brecevic E Vincent M Dar R. The Role of Surgery and Postoperative Chemoradiation Therapy in Patients With Lymph Node Positive Esophageal Carcinoma. Cancer (2001) 91:2423–30. 10.1002/1097-0142(20010615)91:12<2423::AID-CNCR1277>3.0.CO;2-1 11413534

[12] Veldeman L Madani I Hulstaert F Meerleer GD Mareel M De Neve W. Evidence Behind Use of Intensity-Modulated Radiotherapy: A Systematic Review of Comparative Clinical Studies. Lancet Oncol (2008) 9:367–75. 10.1016/S1470-2045(08)70098-6 18374290

[13] ICRU. Prescribing, Recording and Reporting Photon Beam Therapy (Report 62) (Supplement to ICRU Report 50). Bethesda, MD: ICRU (1999).

[14] Oppedijk V Van DGA Van Lanschot JJB van Hagen P van Os R van Rij CM. Patterns of Recurrence After Surgery Alone Versus Preoperative Chemoradiotherapy and Surgery in the CROSS Trials. J Clin Oncol (2014) 32(5):367–9. 10.1200/JCO.2013.51.2186 24419108

[15] Goldstein HM Rogers LF Fletcher GH Dodd GD. Radiological Manifestations of Radiation-Induced Injury to the Normal Upper Gastrointestinal Tract. Radiology (1975) 117(1):135–40. 10.1148/117.1.135 1162052

[16] Kachnic LA Winter K Wasserman T Kelsen D Ginsberg R Pisansky TM. Longitudinal Quality-of-Life Analysis of RTOG 94-05 (Int 0123): A Phase III Trial of Definitive Chemoradiotherapy for Esophageal Cancer. Gastrointest Cancer Res (2011) 4:45–52. 21673875 PMC3109887

[17] Cooper JS Guo MD Herskovic A Macdonald JS Martenson JA Jr. Al-Sarraf M. Chemoradiotherapy of Locally Advanced Esophageal Cancer: Long-Term Follow-Up of a Prospective Randomized Trial (RTOG 85-01). Radiation Therapy Oncology Group. JAMA (1999) 281:1623–7. 10.1001/jama.281.17.1623 10235156

[18] Schreiber D Rineer J Vongtama D Wortham A Han P Schwartz D. Impact of Postoperative Radiation After Esophagectomy for Esophageal Cancer. J Thoracic Oncol (2010) 5(2):244–50. 10.1097/JTO.0b013e3181c5e34f 20009774

[19] Ando N Iizuka T Ide H Ishida K Shinoda M Nishimaki T. Surgery Plus Chemotherapy Compared With Surgery Alone for Localized Squamous Cell Carcinoma of the Thoracic Esophagus: A Japan Clinical Oncology Group Study—Jcog9204. J Clin Oncol 21(24). 10.1200/JCO.2003.12.095 14673047

