# Supplementary material for: The Prognosis and Feasibility of Extensive Clinical Target Volume in Postoperative Radiotherapy for Esophageal Squamous Cell Carcinoma: A Phase II Clinical Trial
Source: Front Oncol. 2021 Jul 2;11:669575. doi: 10.3389/fonc.2021.669575 (PMC8291030; doi:10.3389/fonc.2021.669575)
Supplement: Supplementary file 1 [file DataSheet_1.docx]

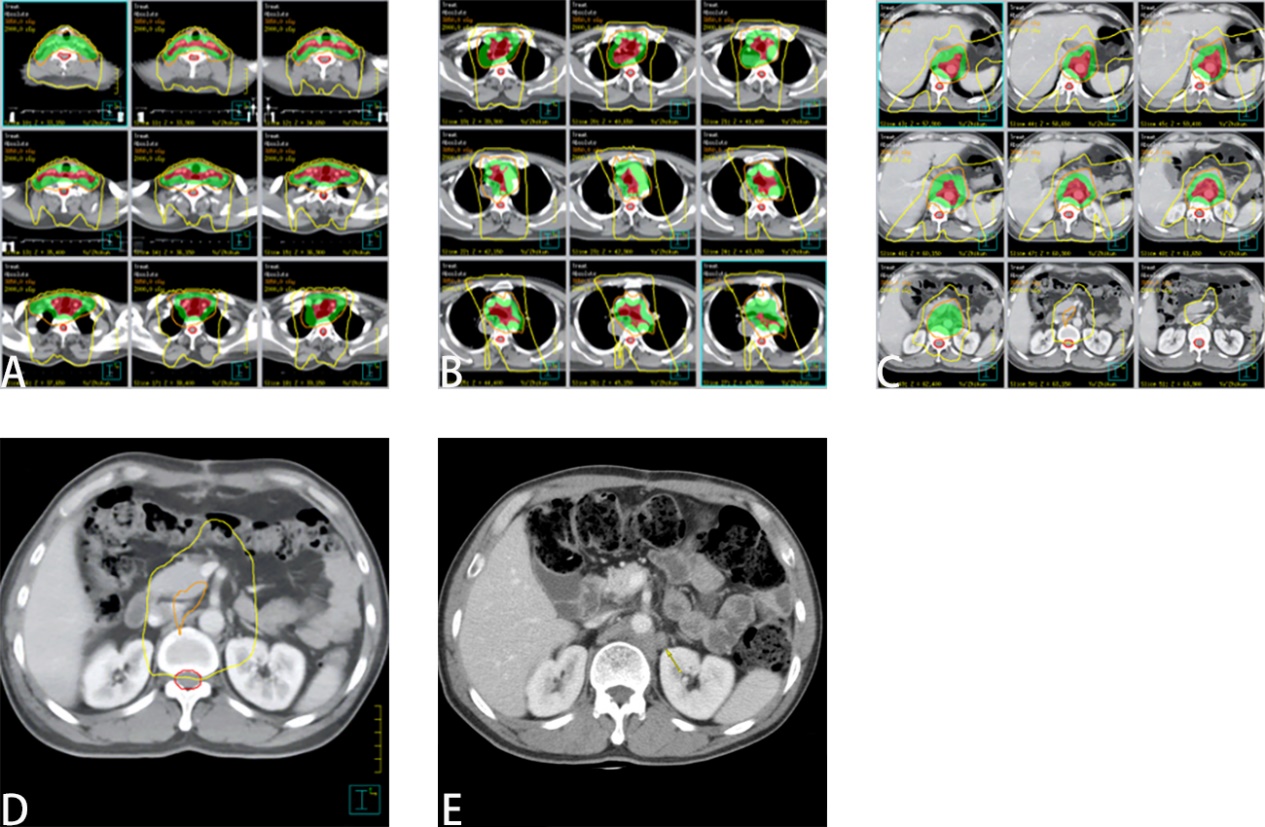


Figure supplement 1 shows the only patient with marginal recurrence. ABC is the display of the whole target area, yellow represents 50% isodose line, orange represents 95% isodose line, red shadow represents CTV, green negative represents PTV, and E is the CT image of recurrence.


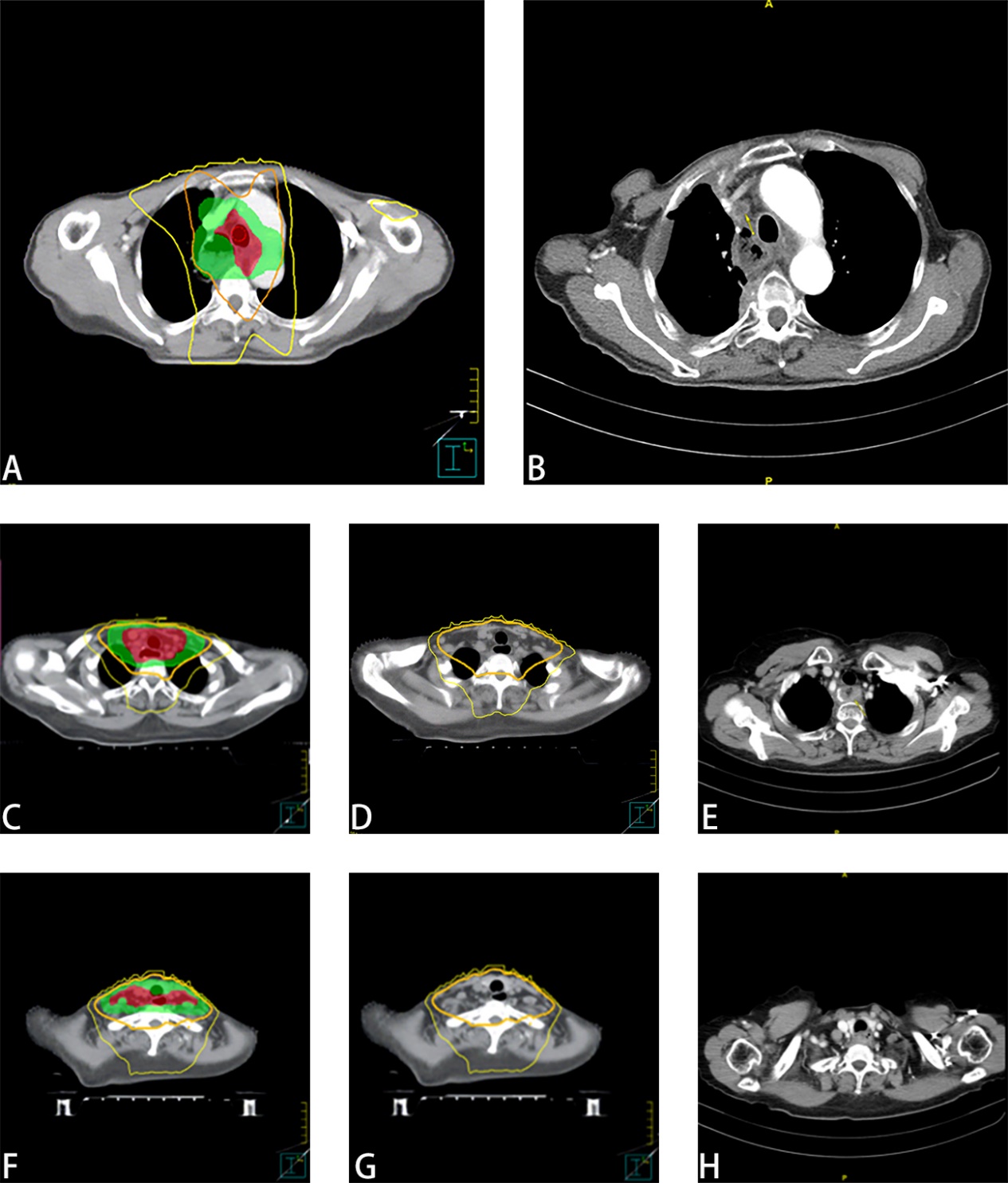


Figure supplement 2 shows cases of recurrence in the field. A and B showed CT images of recurrence in the mediastinal infield. C. D and E showed CT images of recurrence in the anastomotic field. F. G and h showed the CT image of recurrence in supraclavicular region
