# Supplementary material for: The Prognosis and Feasibility of Extensive Clinical Target Volume in Postoperative Radiotherapy for Esophageal Squamous Cell Carcinoma: A Phase II Clinical Trial
Source: Front Oncol. 2021 Jul 2;11:669575. doi: 10.3389/fonc.2021.669575 (PMC8291030; doi:10.3389/fonc.2021.669575)
Supplement: Supplementary file 2 [file Table_1.docx]

Table Supplement1: Normal Organ Dose Restrictions

| Risk organ | Contour regulation | Dose restriction |
| --- | --- | --- |
| Spinal cord | All the layers of CT scan have to be contoured and the margin of vertebra tube can be regarded as that of planning organ at risk volume. | Highest point dose less than 45Gy |
| Lung | It is allowed to use automatic tools in the delineation of margin of lungs. (Trachea and bronchia must be contoured manually) | V20 of lung (PTV excluded) has to be less than 25%, V5 less than 65% and the mean dose has to be less than 13Gy. |
| Heart | The superior margin of heart consists of right atrium and right ventricle, pulmonary artery trunk, ascending main aorta and superior vena cava excluded. The inferior margin is at the level of heart apex. | V30 of heart has to be less than 45% and the mean dose has to be less than 36Gy. |

Table Supplement2: Dose limit exceeded rate

| Risk organ | Dose restriction | Dose limit  exceeded rate（%） |
| --- | --- | --- |
| Spinal cord | Highest point dose less than 45Gy | 6.5 |
| Lung | V20 has to be less than 25% | 71.7 |
|  | V5 less than 65% | 52.2 |
|  | the mean dose has to be less than 13Gy. | 10.9 |
| Heart | V30 of heart has to be less than 45% | 87.0 |
|  | the mean dose has to be less than 36Gy. | 2.2 |
|  | the mean dose has to be less than 27Gy. | 89.1 |

Table Supplement3: Patterns of First Treatment Failure Comparison

|  | S team  in Xiao study | S and R team  in Xiao study | Our study |
| --- | --- | --- | --- |
| Intrathoracic lymph node metastasis | 63(25%) | 31(16.2%) | 15(22.86%) |
| Anastomotic recurrence | 14(5.8%) | 1(0.5%) | 5(7.14) |
| Supraclavicular lymph node metastasis | 38(13.2%) | 6(3.1%) | 10(14.28%) |
| Intraabdominal metastasis | 24(9.9%) | 14(7.3%) | 3(4.29) |
| Hematogenous metastasis | 44(18.1%) | 45(23.6%) | 18(25.7) |

S: surgery alone team;S and R: surgery plus radiotherapy team.
